# Two new species of the millipede family Cambalopsidae from Myanmar (Diplopoda, Spirostreptida)

**DOI:** 10.3897/zookeys.760.24837

**Published:** 2018-05-28

**Authors:** Natdanai Likhitrakarn, Sergei I. Golovatch, Ruttapon Srisonchai, Franck Brehier, Aung Lin, Chirasak Sutcharit, Somsak Panha

**Affiliations:** 1 Division of Plant Protection, Faculty of Agricultural Production, Maejo University, Chiang Mai, 50290, Thailand; 2 Institute for Problems of Ecology and Evolution, Russian Academy of Sciences, Leninsky pr. 33, Moscow 119071, Russia; 3 Animal Systematics Research Unit, Department of Biology, Faculty of Science, Chulalongkorn University, Bangkok, 10330, Thailand; 4 09800 Alas, Balaguères, France; 5 Fauna & Flora International, Myanmar Program, Myay Nu Street, Sanchaung Township, Yangon, Myanmar

**Keywords:** diplopod, key, map, Myanmar, new species, *Plusioglyphiulus*, *Trachyjulus*

## Abstract

Two new species of cave-dwelling millipedes are described from Myanmar, one each in the genera *Plusioglyphiulus* Silvestri, 1923 and *Trachyjulus* Peters, 1864. *Plusioglyphiulus digitiformis* **sp. n.** joins the small peculiar group of congeners from Thailand which is characterized by such plesiomorphies as the tergal crests on the collum and following metaterga being transversely divided into two, not three parts, as in species of the genus *Glyphiulus* Gervais, 1847. However, this new species differs by the 3-segmented telopodites of ♂ legs 1, the anterior gonopodal coxosternum showing higher and nearly straight apicomesal processes and very evident apicolateral teeth, as well as the higher and acuminate paramedian coxal processes of the posterior gonopods, the latter’s telopodites demonstrating an apical fovea bearing a group of microsetae at the bottom. *Trachyjulus bifidus* **sp. n.** is primarily distinguished by the telopodites of their anterior gonopods being strikingly and deeply bifid. A key to the five species of Cambalopsidae currently known to occur in Myanmar is presented, and a map showing their distributions given.

## Introduction

Myanmar is globally recognized as a highly important hotspot of biodiversity, supporting a great number of species and abundant forest resources (Myers et al. 2000). Unfortunately, by 2010 the deforested areas in Myanmar totalled 21,178.8 km^2^, with an annual deforestation rate of 0.81% between 1990 and 2010 (Wang and Myint 2016). Yet some regions, especially montane ones, remain rich in woodlands, including primary tropical forest.

Cambalopsidae is the largest family in the suborder Cambalidea, order Spirostreptida, and it currently contains > 100 species in 7–8 genera, all in Southeast Asia and Indo-Australia, up to central China in the north and Borneo in the east (Hoffman 1980, Mauriès 1983). Only a couple of anthropochore species have attained particularly vast pantropical distributions.

Pocock (1893) was the first to describe cambalopsids from Myanmar, three new species currently referred to as *Trachyjulus calvus* (Pocock, 1893), *Podoglyphiulus doriae* (Pocock, 1893) and *P. feae* (Pocock, 1893). According to the latest catalogue of the Diplopoda of Myanmar (Likhitrakarn et al. 2017), the fauna of that country currently amounts to 92 species, including those first three cambalopsids of Pocock.

After more than 120 years of complete inactivity in this respect, the present paper puts on record another two new species of Cambalopsidae from Myanmar. It also provides a key to all five species of this family in that country, as well as a map showing their distributions. The two new species described below are also the first to come from caves in Myanmar.

## Material and methods

The material was collected in Myanmar in 2015–2016 by Somsak Panha and members of the Animal Systematics Research Unit, Chulalongkorn University, as well as by a French collecting team headed by Louis Deharveng, of the Muséum National d’Histoire Naturelle, Paris, France. Photographs of live animals were taken in the laboratory using a Nikon 700D digital camera with a Nikon AF-S VR 105 mm macro lens. Specimens were preserved in 75% ethanol, and morphological observations made under an Olympus SZX7 stereo microscope.

Scanning electron micrographs (SEM) were taken applying a JEOL, JSM-5410 LV microscope, and the material returned to alcohol upon examination. Pictures of the gonopods of the holotypes were taken in the laboratory and assembled using “Cell^D^” automontage software of the Olympus Soft Imaging Solution package. The key below is primarily based on the descriptions by Golovatch et al. (2007a, 2007b, 2009, 2011). One of the holotypes, as well as most of the paratypes are housed in the Museum of Zoology, Chulalongkorn University (CUMZ), Bangkok, Thailand. The other holotype and several paratypes are stored in the Muséum national d’Histoire naturelle (MNHN), Paris, France, while a few paratypes are deposited in the collection of the Zoological Museum, State University of Moscow (ZMUM), Russia, as indicated in the text.

The collecting sites were located by GPS using the WGS84 datum.

The carinotaxic formulae in the descriptions follow those in Golovatch et al. (2007a, b, 2009, 2011), while body segment counts are after Enghoff (1993).

## Taxonomic part

### Family Cambalopsidae Cook, 1895

#### Genus *Plusioglyphiulus* Silvestri, 1923

##### Plusioglyphiulus digitiformis sp. n.

Taxon classificationAnimaliaSpirostreptidaGlyphiulidae

http://zoobank.org/0F9B6EEB-4144-48BE-B71E-F4422E7AFA13

Figs 1 , 2 , 3 , 4

###### Holotype

♂ (CUMZ), Myanmar, Shan State, Taunggyi, Hopong, Parpant area, cave, 20°43'30"N, 97°08'04"E, 23.09.2015, leg. C. Sutcharit and R. Srisonchai.

###### Paratypes.

7 ♂, 18 ♀ (CUMZ), same data as holotype. 2 ♂, 1 ♀ (MNHN, MY15-16/09), Shan State, Jatwet Gu (Linwe Depression Cave #2), limestone, 21°13'40"N, 96°33'24"E, 29.11.2015; 1 ♀ (MNHN, MY15-17/10 (SS06)), same State, Kyauk Khaung Cave (Stone Cave), limestone, 21°11'28"N, 96°33'09"E, 29.11.2015; 9 ♂, 17 ♀, 1 juv. (MNHN, MY15-18/06), same State, Mondawa Gu Cave, limestone, 20°45'17"N, 97°01'03"E, 01.12.2015; 7 ♀ (MNHN, SS11), same locality, 21.09.2015; 13 ♂, 15 ♀ (MNHN, MY15-20/04), same State, Parpent Cave n°1, Guano, limestone, 20°51'03"N, 97°14'23"E, 02.12.2015; 2 ♂, 3 ♀, 4 juv. (MNHN, SS15), same locality, 23.09.2015; 6 ♂, 17 ♀ (MNHN, MY15-21/07), same State, Parpent Cave n°2, Guano, limestone, 20°51'04"N, 97°14'28"E, 02.12.2015, all leg. F. Bréhier.

###### Other material.

4 ♂, 14 ♀ (MNHN, MY15-14/09), 2 ♂, 3 ♀ (ZMUM), Mon State, Saddan Sin Gu Cave, limestone, tower karst, 16°31'43"N, 97°43'02"E, 26.11.2015; 1 juv. (MNHN, MY15-15/07), same State, Nathack Gu Cave (Two Level Cave), limestone, tower karst, 16°31'33.5"N, 97°42'48.8"E, 26.11.2015, all leg. F. Bréhier.

###### Etymology.

To emphasize the finger-shaped apicomesal coxoternal processes (**acp**) of the anterior gonopodal coxosternum; adjective.

###### Diagnosis.

This new species is apparently most similar to *P. antiquior* Golovatch, Geoffroy, Mauriès & VandenSpiegel, 2011, from a cave in Kanchanaburi Province, Thailand (Golovatch et al. 2011), in sharing the special the carinotaxic formulae of the collum and postcollum rings (Fig. 2A–C, H, I, N, O), ♂ legs 1 with a short central hook (Figs 3A, B, 4C), ♂ legs 2 with modestly enlarged telopodites (Figs 3D, 4D), coupled with the simple plate-like anterior gonopods (Figs 3G, H, 4G), the complex posterior gonopods in which the coxites are densely setose paramedially and each supplied with an evident fovea, and the telopodites are evident and digitiform (Figs 3K, L, 4H, I). However, the new species differs from *P. antiquior* in the more clearly divided crests on metaterga, the lateral ones being somewhat higher, coupled with the 3-segmented telopodites of ♂ legs 1, the anterior gonopodal coxosternum showing higher and nearly straight apicomesal coxoternal processes (acp) and very evident basolateral coxosternal processes (bcp), as well as the higher and acuminate anterior coxal processes (ap) of the posterior gonopods, the latter’s telopodite (te) demonstrating an apical fovea that bears a group of microsetae at the bottom (Figs 3K, L, 4H, I).

###### Description.

Length of holotype ca. 18 mm; adult paratypes 12–27 (♂) or 13–29 mm (♀); midbody segments circular in cross-section (Fig. 2K), width in holotype 0.9 mm; paratypes 0.8–1.0 (♂, ♀).

**Figure 1.**
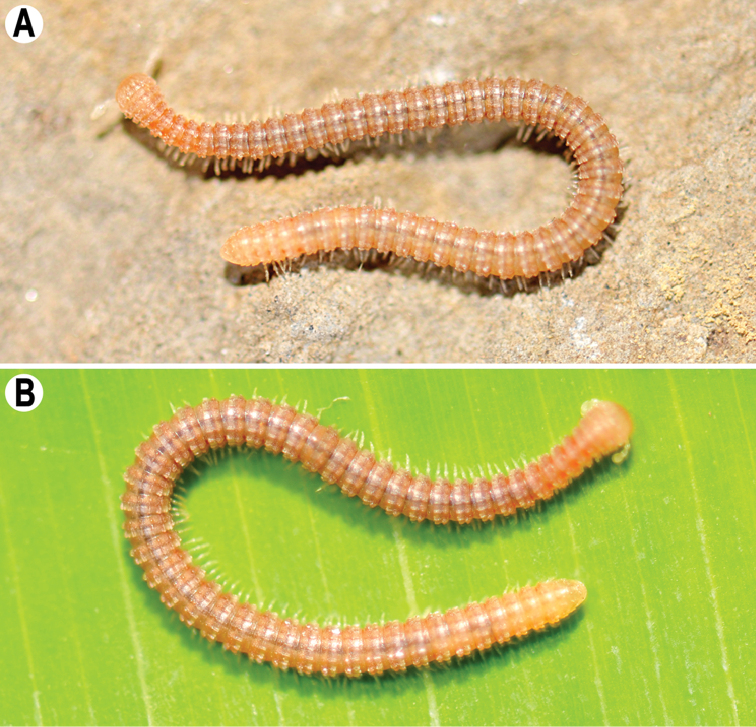
*Plusioglyphiulus digitiformis* sp. n., **A, B**, ♀ paratype from Parpant area, live animal. Pictures by R. Srisonchai, not to scale.

Colouration of live animals light red-brown (Fig. 1) with lighter anterior and posterior parts of body; antennae, venter and legs light yellowish; coloration in alcohol, after two years of preservation, uniformly light red brownish to dark castaneous brown, dorsal crests and porosteles usually dark brownish. Antennae and venter yellow brownish to brownish. Ommatidia brown to blackish.

**Figure 2.**
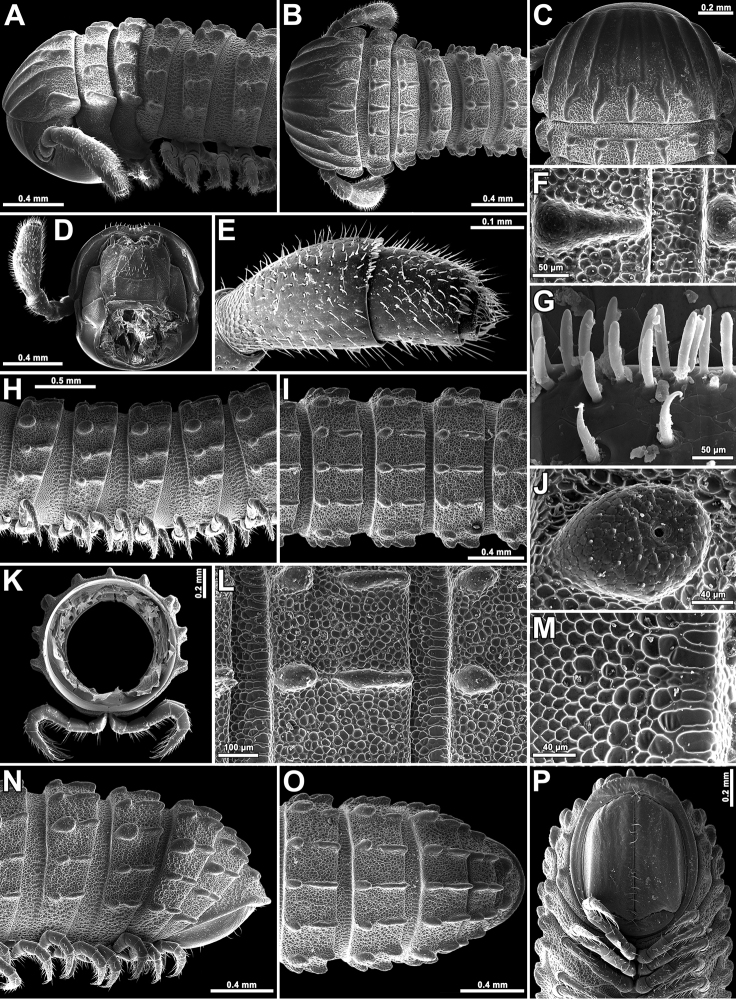
*Plusioglyphiulus digitiformis* sp. n., **A–C, F, H–J, L, M** ♀ paratype from Parpant area **D, E, G, K, N–P** ♂ paratype from Parpant area. **A, B** anterior part of body, lateral and dorsal views, respectively **C** collum and body ring 2, dorsal view **D** head, ventral view **E** anterior part of antenna, lateral view **F** second body crest, dorsal view **G** bacilliform sensilla on antennomere 5, lateral view **H, I** midbody rings, lateral and dorsal views, respectively **J** porostele, lateral view **K** cross-section of a midbody segment **L** midbody crests, dorsal view **M** midbody prozona, dorsal view **N–P** posterior part of body, lateral, dorsal and ventral views, respectively.

Adult body with 46p+3a+T (holotype); paratypes with 37–60p+1–4a+T (♂) or 36–66p+1–4a+T (♀). Eye patches transversely ovoid, with 3+(1–2) blackish, rather flat ommatidia in 1–2 longitudinal rows. Antennae short and clavate (Figs 1, 2A, 2B, 2D, 2E, 4A), extending behind ring 4 laterally, antennomeres 5 and 6 each with a small apicodorsal field or corolla of bacilliform sensilla (Figs 2E, 4A). Gnathochilarium oligotrichous, each lamella lingualis with 3–4 setae; mentum undivided (Fig. 4B).

In width, collum = midbody rings (close to 13^th^ to 15^th^) > head = ring 4 > 10 > 9 > 8 > 7 > 6 > 4 = 5 > 2 > 3; body abruptly tapering towards telson on a few posteriormost rings (Fig. 2N–P). Postcollar constriction evident due to only a moderately enlarged collum (Fig. 2B, C).

Collum with 6+6 longitudinal crests starting from anterior edge, carinotaxic formula of collum, 1+2p+3+4p/t+5p/t+pp+/ma (Fig. 2A–C).

Following metaterga similarly strongly crested (Figs 1, 2A–C, H, I, L, N, O), especially so from ring 5 onwards, whence porosteles commence, these tubercles clearly reduced on legless segments where ozopores are missing (Fig. 2N). Porosteles large, high, conical, round, directed caudolaterad, wider than high (Fig. 2J); ozoporiferous crests distinctly divided into two about midway, their anterior halves being higher (Fig. 2A, B, C, H, I, L, N, O). Carinotaxic formulae of metaterga 2–4, 2+2/2+M+2/2+2 (Fig. 2A, B); usual formula of following metaterga, 2/2+I/i+3/3+I/i+2/2 (Fig. 2A, B, H, I, L, N, O); all crests and tubercles low.

Tegument delicately alveolate-areolate (Fig. 2B, H, I, L, M, N, O), dull throughout. Fine longitudinal striations in front of stricture between pro- and metazonae, remaining surface of prozonae very delicately shagreened (Fig. 2F, L). Metatergal setae absent. Segments 2 and 3 each with long pleural flaps.

Limbus extremely finely and more or less regularly denticulate.

Epiproct (Fig. 2N–P) broadly rounded apically, with 1+1 paramedian tubercles at midway. Paraprocts rather clearly flattened, each with a faint premarginal sulcus medially (Fig. 2P). Hypoproct emarginated at caudal margin (Fig. 2P)

Ventral flaps behind gonopod aperture on ♂ segment 7 barely distinguishable as low swellings, forming no marked transverse ridge.

Legs nearly as long as body diameter (Fig. 2K), claw at base with a strong accessory claw almost half as long as claw itself (Fig. 4F).

**Figure 3.**
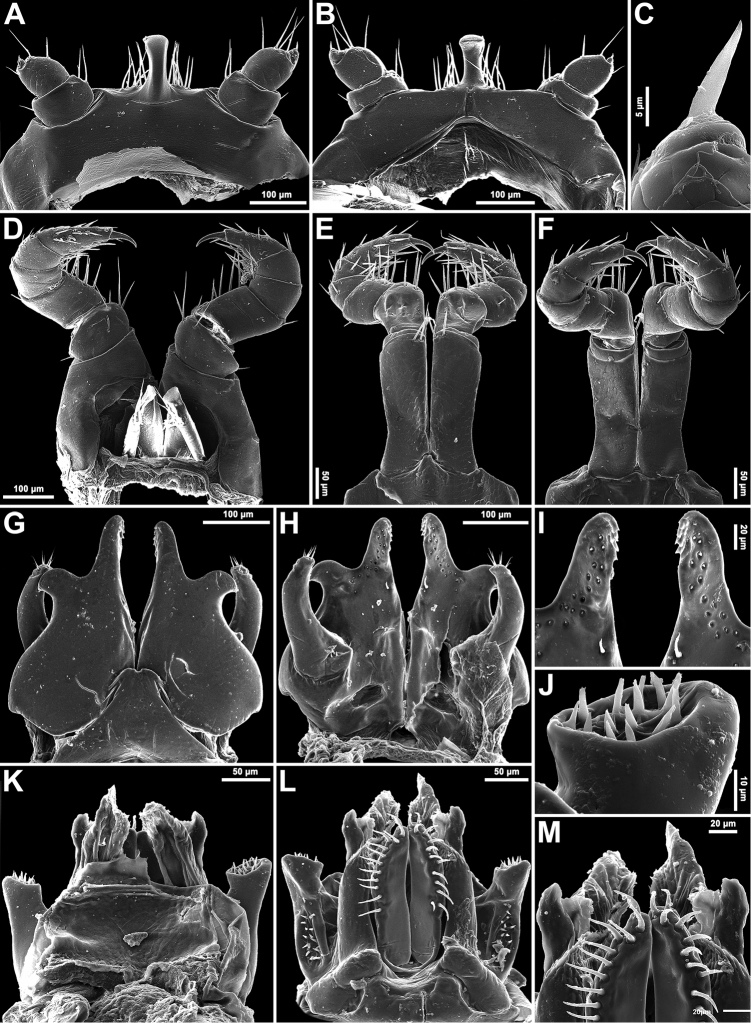
*Plusioglyphiulus digitiformis* sp. n., ♂ paratype from Parpant area. **A, B** ♂ legs 1, caudal and anterior views, respectively **C** claw of ♂ leg 1, anterior view **D** ♂ legs 2, caudal view **E, F** ♂ legs 3, anterior and caudal views, respectively **G, H** anterior gonopods, anterior and caudal views, respectively **I** microsetae on top of coxal processes of anterior gonopods, caudal view **K, L** posterior gonopods, caudal and anterior views, respectively **J** tip of telopodite of posterior gonopod, caudal view **M** setose lobe on telopodite of posterior gonopod, anterior view.

**Figure 4.**
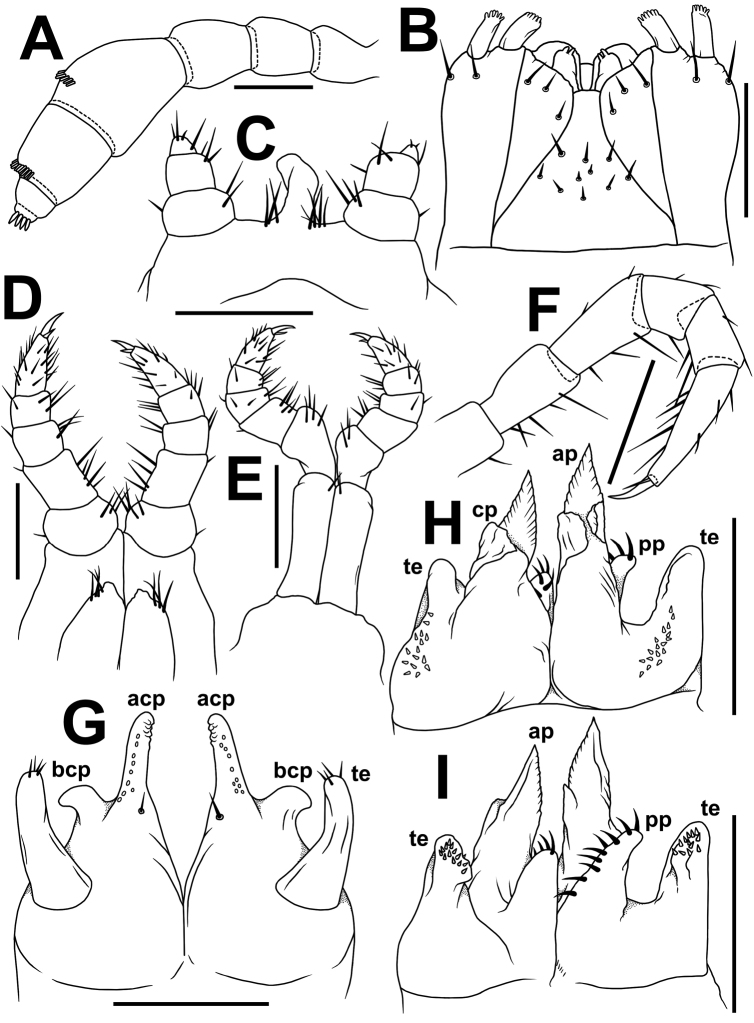
*Plusioglyphiulus digitiformis* sp. n., **A, B** ♂ paratype from Mondawa Gu Cave **C–H** ♂ paratype from Parpent Cave n°2. **A** antenna, lateral view **B** gnathochilarium, ventral view **C** legs 1, anterior view **D** legs 2, caudal view **E** legs 3, caudal view **F** midbody leg, anterior view **G** anterior gonopods, caudal view **H, I** posterior gonopods, caudal and anterior views, respectively. Abbreviations: **acp** apiconmesal coxoternal process **bcp** basolateral coxosternal process **te** telopodites **ap** anterior coxal processes **cp** caual coxal processes **pp** paramedian coxal processes. Scale bars: 0.2 mm.

♂ legs 1 with an unusually short, central hook and relatively strongly reduced, 3-segmented telopodites (Figs 3A, B, 4C), each with a small and sharp claw (Fig. 3C).

♂ legs 2 clearly enlarged, with high and large coxae; telopodites hirsute on anterior face; penes broad, oblong-subtrapeziform, fused at base, each with 3–4 strong setae distolaterally (Figs 3D, 4D).

♂ legs 3 modified in having coxae especially slender and elongate, but with somewhat shortened telopodites (Figs 3E, F, 4E).

Anterior gonopods (Figs 3G–I, 4G) with a typical shield-like coxosternum, the latter modestly setose on caudal face and provided with a concave notch separating a pair of high, nearly straight, terminally rounded, apicomesal, coxosternal processes (acp) and a much lower basolateral coxosternal processes (bcp), these being rounded at tip; telopodite (te) typical, rather stout, movable, 1-segmented, lateral in position, with several strong apical setae and a field of small microsetae at base, slightly longer than adjacent bcp.

Posterior gonopods (Figs 3J–M, 4H, I) highly compact, contiguous basally until about midheight; each with a densely setose paramedian coxal process (pp) (Fig. 3M) and with two higher central pieces: anterior coxal process (ap) elongate, distally represented by an acuminate lamina; caudal coxal process (cp) subtriangular, membranous, rounded at tip; each telopodite (te) vase-shaped, with a compact group of coniform microsetae placed at bottom of an apical fovea (Fig. 3J), with another, parabasal field of microsetae on anterior face (Figs 3L, 4H, I).

###### Remarks.

The genus *Plusioglyphiulus* Silvestri, 1923 has recently been reviewed (Golovatch et al. 2009, 2011) and shown to comprise 27 species ranging from northern Thailand and Laos in the west to Borneo in the east and southeast. This new species is the first *Plusioglyphiulus* to be recorded from Myanmar. Based on the pigmented body and eye patches, and like most if not all other cave-dwelling congeners known to date, *P. digitiformis* sp. n. seems to be hardly more than a troglophile.

Most species of this genus show particularly enlarged colla with the tergal crests both on the collum and following segments being clearly divided transversely into three parts. Only two species, *P. antiquior* and *P. panhai* Golovatch, Geoffroy, Mauriès & VandenSpiegel, 2011, both from caves in Thailand and both found quite close to the frontier to Myanmar, are remarkable in still showing the pattern of carinotaxy observed in the genus *Glyphiulus* Gervais, 1847 (Golovatch et al. 2011).

In particular, while their gonopods are relatively complex and unequivocally the same as in typical *Plusioglyphiulus*, the carinotaxic pattern is simple and typical of *Glyphiulus*, i.e., the crests on their colla and following metaterga are divided transversely into two, not three, parts. In this respect, *P. digitiformis* sp. n. clearly joins the above duet, showing the closest similarities, both morphologically and geographically, to *P. antiquior*.

Non-type material shows all characters of the type series, but their localities lie very far from the others (ca. 470 km) (Fig. 8). We hope that future molecular studies will answer the question of the conspecificity (or not) of all above populations.

Interestingly, the famous Burmese amber, 99–100 Mya, appears to contain a typical *Plusioglyphiulus* yet to be described (Wesener in litt.). This is evidence both of the very old age of this genus and its long presence *in situ*.

#### Genus *Trachyjulus* Peter, 1864

##### Trachyjulus bifidus sp. n.

Taxon classificationAnimaliaSpirostreptidaGlyphiulidae

http://zoobank.org/73DE8D2F-8205-4CC9-9B72-8F802F569454

Figs 5 , 6 , 7

###### Holotype

♂ (CUMZ), Myanmar, Tanintharyi Region, San Gu Cave (Elephant Cave), limestone, tower karst, 11°13'55"N, 99°10'32"E, 17.11.2015, leg. F. Bréhier.

###### Paratypes.

3 ♂, 1 ♀, 3 juv. (CUMZ), 1 ♂, 3 juv. (MNHN, MY15-01/01), same data as holotype. 6 ♂, 7 ♀, 4 juv. (MNHN, MY15-02/27), 2 ♂, 2 ♀ (ZMUM), same Region, Yae Gu Cave (River Cave), limestone, tower karst, 11°13'05"N, 99°10'32"E, 21.11.2015; 12 ♂, 10 ♀, 5 juv. (MNHN, MY15-07/13), same Region, Linno Gu n°1 Cave, guano, limestone, tower karst, 76 m a.s.l., 11°13'35"N, 99°10'32"E, 19.11.2015, all leg. F. Bréhier. 3 ♂, 2 ♀ (MNHN, MY15-09), same Region, Thin Bow Gu Cave (Linno Gu #2), limestone, tower karst, 11°11'23"N, 99°10'18"E, 03.06.2015, leg. C. Rahmadi.

###### Etymology.

To emphasize the strongly bifid telopodites of the anterior gonopods; adjective.

###### Diagnosis.

Differs from other *Trachyjulus* species based primarily on the following combination characters: the strongly elongated and bifid telopodites (te) of the anterior gonopods, coupled with the absence of flagella and the presence of deeply bipartite posterior gonopods, in which the telopodites (te) are much shorter than the massive, paramedian, coxal processes (cp).

###### Description.

**Figure 5.**
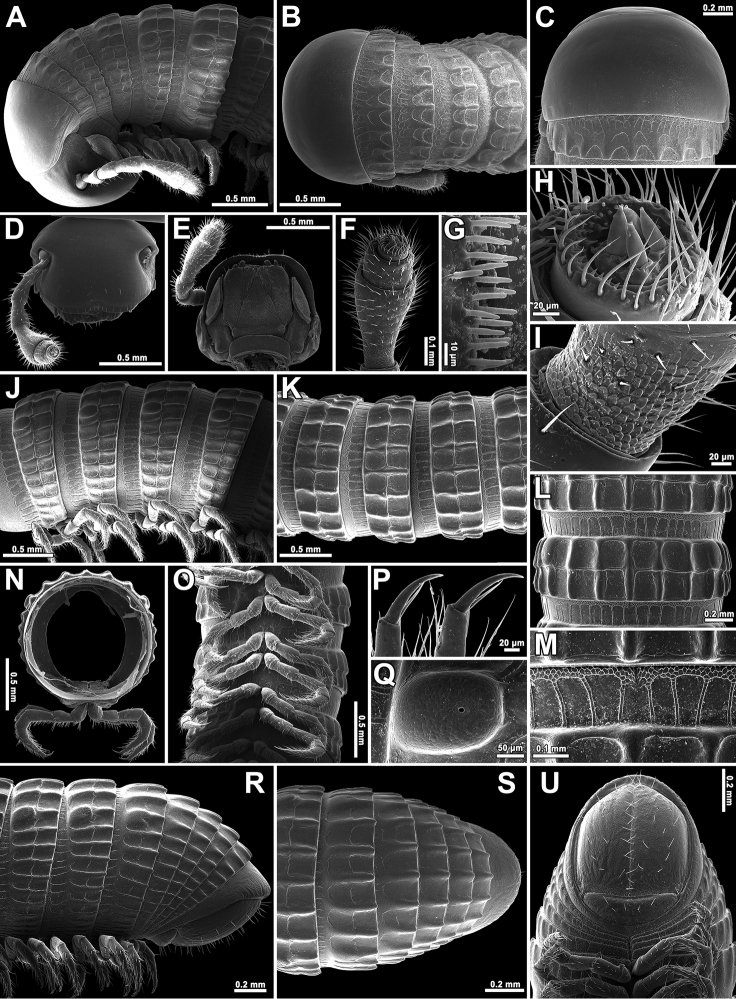
*Trachyjulus bifidus* sp. n., **A–C** ♀ paratype from Linno Gu n°1 Cave **D–U** ♂ paratype from Linno Gu n°1 Cave. **A, B** anterior part of body, lateral and dorsal views, respectively **C** collum and body ring 2, dorsal view **D, E** head, anterior and ventral views **F** anterior part of antenna, ventral view **G** bacilliform sensilla on antennomere 5, lateral view **H** tip of antenna **I** base of antennomere 5, lateral view **J, K, O** midbody rings, lateral, dorsal and ventral views, respectively **L** midbody ring, dorsal view **M** midbody prozona, dorsal view **N** cross-section of a midbody ring **P** claws of midbody legs **Q** midbody porostele, dorsal view **R–U**, posterior part of body, lateral, dorsal and ventral views, respectively.

Length of holotype ca. 19 mm; adult paratypes 13–30 (♂) or 12–25 mm (♀); midbody segments circular in cross-section (Fig. 5N), width of holotype 1.0 mm, of paratypes 0.8–1.0 (♂) or 0.8–1.1 mm (♀).

Coloration of adults in alcohol light grey-brown to dark castaneous brown, without a clear-cut pattern. Head, antennae and venter light yellowish to brownish. Ommatidia brown to blackish.

Adult body with 45p+4a+T (holotype); paratypes with 39–70p+2–4a +T (♂) or 40–60p+2–6a+T (♀). Eye patches transversely ovoid, with 3(4)+3(1) blackish, rather flat ommatidia in 1–2 longitudinal rows. Antennae short and clavate (Figs 5A, B, D, E, 7A), extending behind segment 5 laterally (Fig. 5A), antennomeres 5–7 each with a small apicodorsal group or corolla of bacilliform sensilla (Figs 5A, F, G, H, 7A), surface at base of antennomere 5 very finely scaly (Fig. 5F, I). Gnathochilarium (Figs 5E, 7B) oligotrichous, each lamella lingualis with 3–4 setae; mentum single.

In width, collum = midbody rings (close to 6^th^ to 8^th^) > head = ring 2 > 8–10 > 7 > 6 > 5 > 3 = 4; body abruptly tapering towards telson on a few posteriormost rings (Fig. 5R, S).

Collum (Fig. 5A–C) smooth, only near lateral edge with 1–3 light, short, superficial striae (Fig. 5A). Postcollar metaterga clearly, but not particularly strongly carinate (Figs 5A, B, J, K, L, R, S), especially so from segment 5 on, whence porosteles commence, these becoming completely absent from legless segments where ozopores are missing (Fig. 5R). Porosteles large, but low, conical, round, directed caudolaterad, wider than high (Fig. 5Q). Carinotaxic formula of metaterga 2–4, 7/7+m/m+7/7 (Fig. 5A–C). Carinotaxic formulae of following segments typically 10–7/10–7+I/i+2/2+m/m (Fig. 5A, B, C, J, K, L, R, S); all crests and tubercles, including porosteles, low.

Tegument smooth (Fig. 5A, B, J, K, L, R, S), dull throughout. Fine longitudinal striations in front of stricture between pro- and metazonae, remaining surface of prozonae very delicately shagreened (Fig. 5J, K, L, R). Metatergal setae absent. Segments 2 and 3 each with long pleural flaps.

Epiproct (Fig. 5R–U) simple, bare, smooth, regularly rounded caudally. Paraprocts smooth, rather regularly convex and densely setose (Fig. 5U). Hypoproct transversely bean-shaped, slightly concave caudally (Fig. 5U).

Ventral flaps behind gonopod aperture on ♂ segment 7 evident swellings, forming a marked transverse ridge.

Legs nearly as long as body diameter (Fig. 5N), claw with an evident and long accessory claw near base (Fig. 5P), the latter up to ca. 2/3^rd^s the length of claw itself (Fig. 5P).

**Figure 6.**
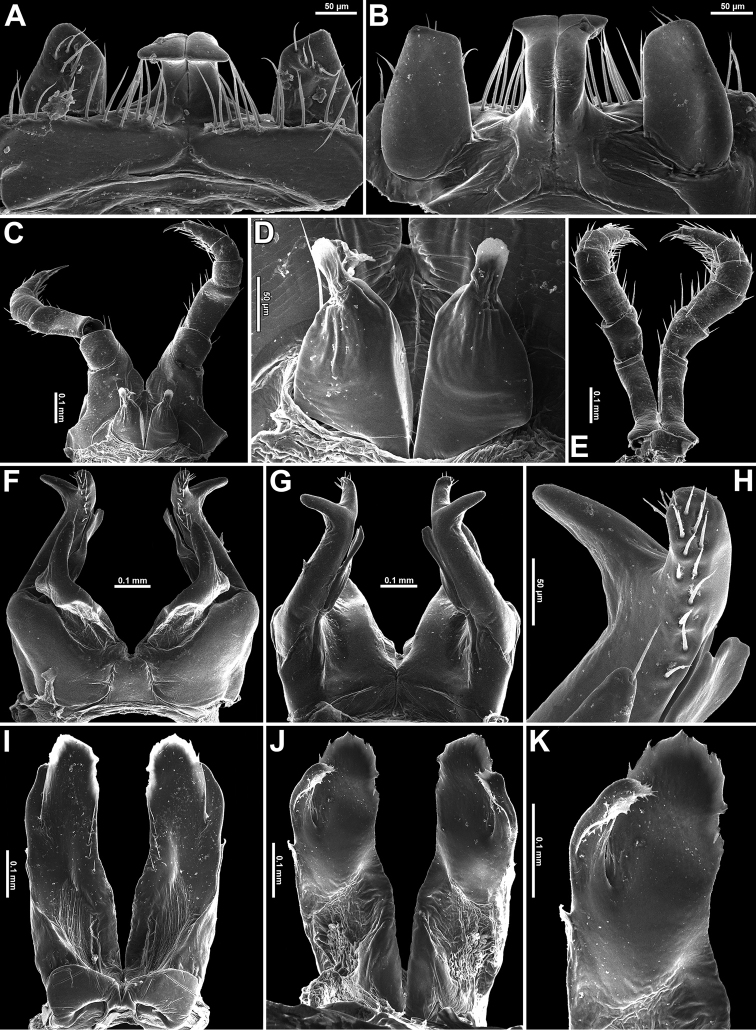
*Trachyjulus bifidus* sp. n., ♂ paratype from Linno Gu n°1 Cave. **A, B** legs 1, anterior and caudal views, respectively **C** leg 2, caudal view **D** penes, caudal view **E** legs 3, caudal view **F, G** anterior gonopods, anterior and caudal views, respectively **H** tip of telopodite of anterior gonopod, caudal view **I, J** posterior gonopods, anterior and caudal views, respectively **K** right posterior gonopod, caudal view.

**Figure 7.**
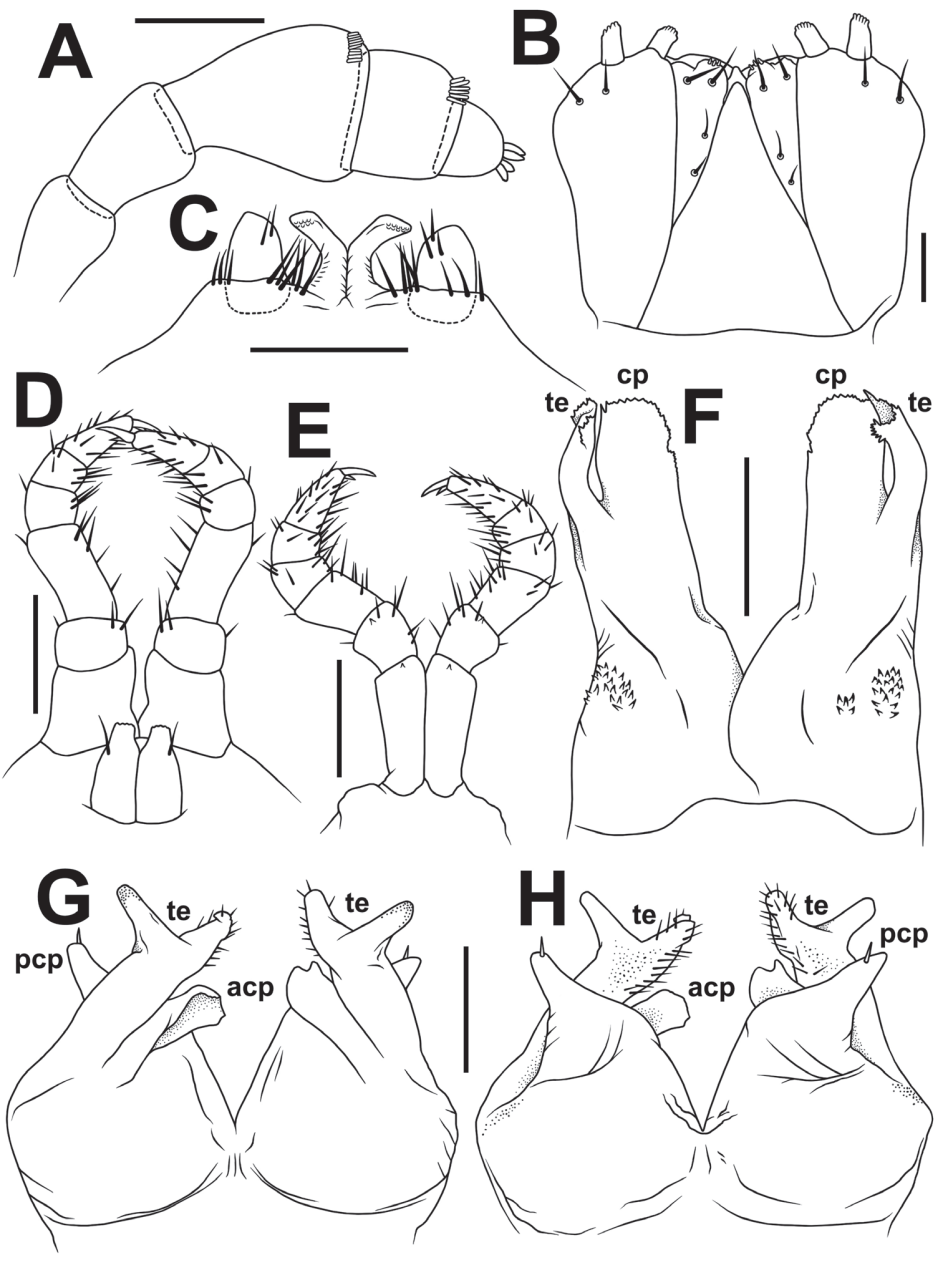
*Trachyjulus bifidus* sp. n., ♂ holotype from San Gu Cave. **A** antenna, lateral view **B** gnathochilarium, ventral view **C** legs 1, anterior view **D** legs 2, caudal view **E** legs 3, caudal view **F** posterior gonopods, caudal view **G H** anterior gonopods, anterior and caudal views, respectively. Abbreviations: **cp** coxal processes **te** telopodites **acp** anterior coxosternal process **pcp** posterior coxoternal process. Scale bars: 0.2 mm.

♂ legs 1 highly characteristic (Figs 6A, B, 7C) in being very strongly reduced, with large 1-segmented telopodites and a pair of large, hook-shaped, medially contiguous, sternal processes with groups of long and strong setae at base on caudal face.

**Figure 8.**
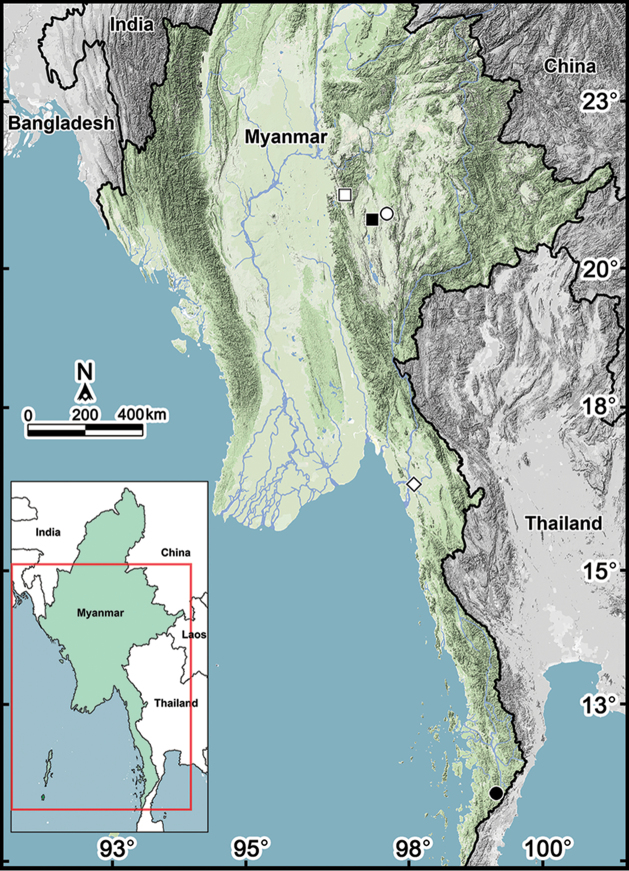
Distribution of two new cambalopsid species. Key: □ *Plusioglyphiulus digitiformis* sp. n., Jatwet Gu and Kyauk Khaung Cave ■ *Plusioglyphiulus digitiformis* sp. n., Mondawa Gu Cave △ *Plusioglyphiulus digitiformis* sp. n., Cave in Parpant area ● *Plusioglyphiulus digitiformis* sp. n., Parpent Cave n°1 and Parpent Cave n°2 ◊ *Plusioglyphiulus digitiformis* sp. n., Saddan Sin Gu Cave and Nathack Gu Cave ○ *Trachyjulus bifidus* sp. n., San Gu Cave, Yae Gu Cave, Linno Gu n°1 Cave and Thin Bow Gu Cave.

♂ legs 2 slightly reduced, but coxa and femur hypertrophied (Figs 6C, 7D); penes rather small, oblong-subtrapeziform, each with 1–2 strong setae distolaterally (Figs 6C, D, 7D).

♂ legs 3 slightly reduced, modified in having coxae especially slender and elongate (Figs 6E, 7E).

Anterior gonopods (Figs 6F–H, 7G, H)) peculiar in stout telopodites (te) being two curved, widely separated fingers with a setose central field on anterior face (Figs 6F, H, 7H). Anterior coxosternal process (acp) lobe-shaped, caudally about as high as a stout posterior coxosternal process (pcp).

Posterior gonopods (Figs 6I–K, 7F) elongate and finger-shaped, membranous, evidently bipartite, round, with both coxal processes (cp) and telopodites (te) sparsely microspiculate at margin (Fig. 6K); te membranous, slightly curved mesad, clearly shorter than cp, with a parabasal field of coniform microsetae caudally (Figs 6J, 7H).

###### Remarks.

The genus *Trachyjulus* Peters, 1864 is currently known to comprise 31 species ranging from Nepal, India, and Sri Lanka in the west, through Bangladesh and Myanmar to Vietnam, Thailand, Malay Peninsula, Singapore, and Indonesia (Sumatra and Java) in the east (Golovatch et al. 2012). Only one species, the pantropical anthropochore *T. calvus*, has hitherto been documented from Myanmar (Likhitrakarn et al. 2017). This species (cf. Golovatch et al. 2012) is similar to *T. bifidus* sp. n., but the latter is clearly distinguished by the bifid telopodites of the anterior and posterior gonopods.

Based on the pigmented body and eye patches, and like most if not all other cave-dwelling congeners known to date, *T. bifidus* sp. n. seems to be hardly more than a troglophile.

No special key to relevant genera involved seems to be needed, as the one given below to Myanmar species contains the necessary information.

#### Key to Cambalopsidae species currently known to occur in Myanmar, chiefly based on male characters:

**Table:** 

1	Collum smooth, without strong longitudinal crests (Fig. 5A–C)	**2**
–	Collum with strong longitudinal crests (Fig. 2A–C)	**3**
2	Each telopodite (te) of anterior gonopods with a small birdhead-shaped process at tip, coxosternal process slender and undivided	***Trachyjulus calvus* (Pocock, 1893)**
–	Telopodites (te) of anterior gonopods divided into two strongly curved finger-shaped processes (Figs 6F, G, H, 7G, H), coxosternal process also bifid, divided into two processes (Figs 6F, 7G, H)	***Trachyjulus bifidus* sp. n.**
3	♂ legs 1 with 3-segmented telopodites (Figs 3A, B, 4C)	***Plusioglyphiulus digitiformis* sp. n.**
–	♂ legs 1 with 5-segmented telopodites	**4**
4	Carinotaxy of collum: anterior transverse and posterior transverse rows consisting of 10 and 9 crests, respectively	***Podoglyphiulus doriae* (Pocock, 1893)**
–	Carinotaxy of collum: anterior transverse and posterior transverse rows consisting of 6 and 5 crests, respectively	***Podoglyphiulus feae* (Pocock, 1893)**

## Conclusions

There are 94 millipede species currently known to occur in Myanmar, including both new ones described above. The new material comes from some of the 27 caves located within a radius of ca. 70 km around the town of Kalaw, Shan State, northeastern Myanmar (Piccini et al. 2009). Studies on the cave fauna of that country have just begun and there can hardly be any doubt that many more interesting discoveries are ahead. These will certainly concern Diplopoda as well. Because Cambalopsidae are especially diverse and common in karsts of the adjacent parts of China, Laos, Thailand and Malaysia, where they are usually associated with bat guano in caves (Golovatch 2015), the same presumption can easily be extended to the karsts of Myanmar, too.

## Supplementary Material

XML Treatment for Plusioglyphiulus digitiformis

XML Treatment for Trachyjulus bifidus
